# Supplementary material for: Predicting visual function by interpreting a neuronal wiring diagram
Source: Nature. 2024 Oct 2;634(8032):113–23. doi: 10.1038/s41586-024-07953-5 (PMC11446822; doi:10.1038/s41586-024-07953-5)
Supplement: Supplementary file 6 — CRF and ERF predictions for individual TmY4 and TmY9 cells. Analogous to Supplementary Data 3, but for TmY target types. Shown are the top four monosynaptic pathways, the strongest pathway passing through each of the top ten intermediary types (ranking from Extended Data Fig. 7), and the trisynaptic pathway Tm1–TmY–Dm3–TmY (see the section entitled Prediction of spatial normalization). [file 41586_2024_7953_MOESM6_ESM.zip › DataS4/TmY9q/720575940644636183.pdf]

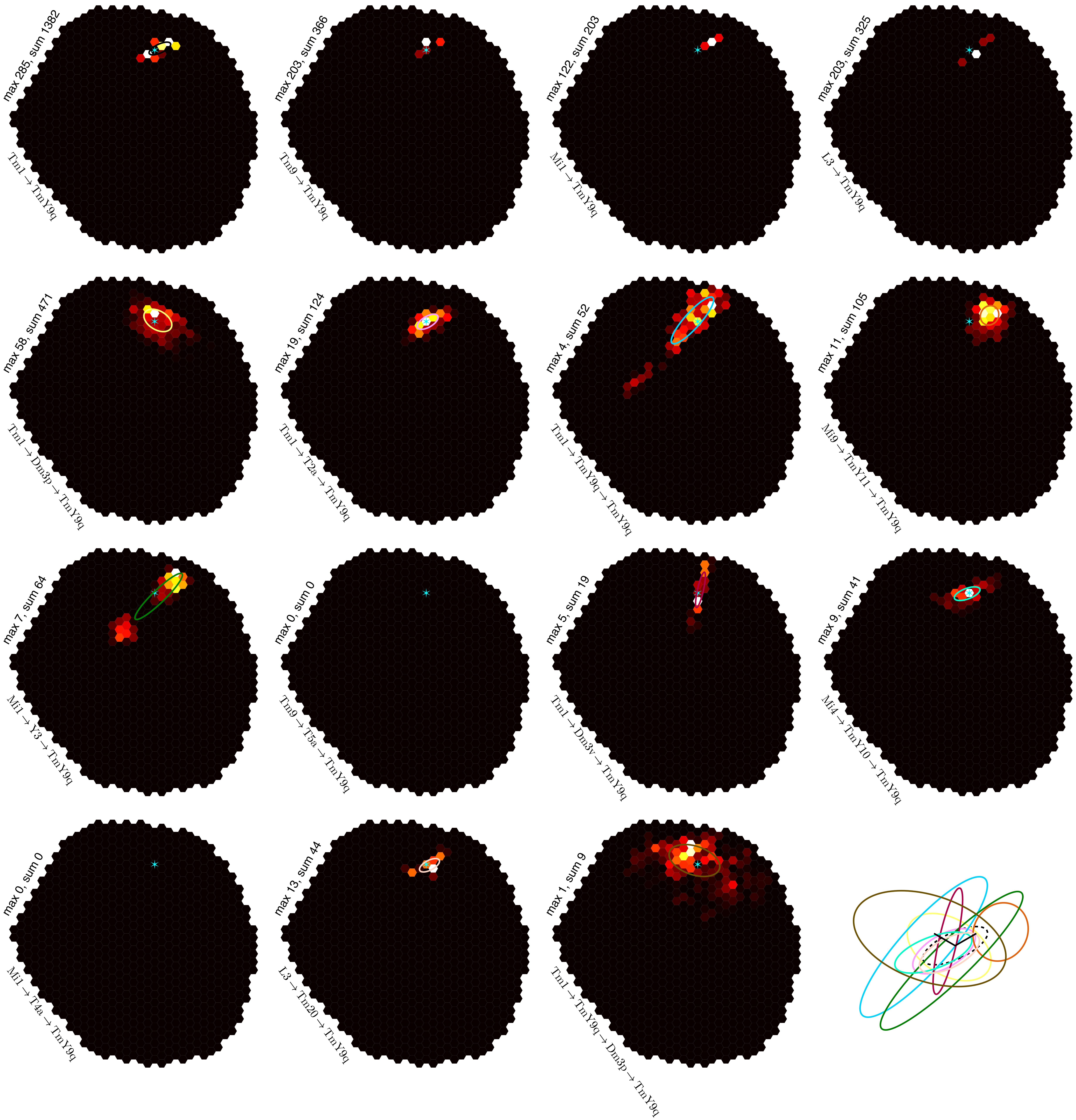
max 285, sum 1382
Tm1 → TmY9q
max 203, sum 366
Tm9 → TmY9q
max 122, sum 203
Mi1 → TmY9q
max 203, sum 325
L3 → TmY9q
max 58, sum 471
Tm1 → Dm3p → TmY9q
max 19, sum 124
Tm1 → T2a → TmY9q
max 4, sum 52
Tm1 → TmY9q → TmY9q
max 11, sum 105
Mi9 → TmY11 → TmY9q
max 7, sum 64
Mi1 → Y3 → TmY9q
max 0, sum 0
Tm9 → T5a → TmY9q
max 5, sum 19
Tm1 → Dm3v → TmY9q
max 9, sum 41
Mi4 → TmY10 → TmY9q
max 0, sum 0
Mi1 → T4a → TmY9q
max 13, sum 44
L3 → Tm20 → TmY9q
max 1, sum 9
Tm1 → TmY9q → Dm3p → TmY9q
